# Nutritional supplements for diabetes sold on the internet: business or health promotion?

**DOI:** 10.1186/1471-2458-13-777

**Published:** 2013-08-26

**Authors:** Loredana Covolo, Michela Capelli, Elisabetta Ceretti, Donatella Feretti, Luigi Caimi, Umberto Gelatti

**Affiliations:** 1 Department of Medical and Surgical Specialties, Radiological Sciences and Public Health, University of Brescia, Viale Europa 11, Brescia 25123, Italy; 2 Post-graduate School of Public Health. University of Brescia, Viale Europa 11, Brescia 25123, Italy; 3 Quality and Technology Assessment, Governance and Communication Strategies in Health Systems” Study and Research Centre - University of Brescia, Viale Europa 11, Brescia 25123, Italy

**Keywords:** Nutritional supplements, Online sales, Diabetes mellitus

## Abstract

**Background:**

Diabetes is one of the most widespread chronic disease. Although many medications are available for the treatment and prevention of diabetes, many people turn to nutritional supplements (NSs). In these years, the online sales have contributed to the growth of use of nutritional supplement. The aim of the research was to investigate the type of information provided by sales websites on NSs, and analyse the existence of scientific evidence about some of the most common ingredients found in available NSs for diabetes.

**Methods:**

A web search was conducted in April 2012 to identify web sites selling NSs in the treatment of diabetes using Google, Yahoo and Bing! and the key word used was “diabetes nutritional supplements”. Website content was evaluated for the quality of information available to consumers and for the presence of a complete list of ingredients in the first NS suggested by the site. Subsequently, in order to analyze the scientific evidence on the efficacy of these supplements a PubMed search was carried out on the ingredients that were shared in at least 3 nutritional supplements.

**Results:**

A total of 10 websites selling NSs were selected. Only half of the websites had a Food and Drug Administration disclaimer and 40% declared clearly that the NS offered was not a substitute for proper medication. A total of 10 NS ingredients were searched for on PubMed. Systematic reviews, meta-analyses or randomized control trials were present for all the ingredients except one. Most of the studies, however, were of poor quality and/or the results were conflicting.

**Conclusions:**

Easy internet access to NSs lacking in adequate medical information and strong scientific evidence is a matter of public health concern, mainly considering that a misleading information could lead to an improper prevention both in healthy people and people suffering from diabetes. There is a clear need for more trials to assess the efficacy and safety of these NSs, better quality control of websites, more informed physicians and greater public awareness of these widely used products.

## Background

Diabetes is one of the most widespread chronic diseases. According to the World Health Organization (WHO), the number of people with diabetes rose from 220 million in 2009 to 346 million in 2011, 90% being diagnosed with diabetes Type 2 (T2D) [1].

Diabetes, is a disease influenced by lifestyle changes, such as diet, and so target of complementary and alternative medicine, including nutritional supplements (NSs) [2].

As defined by Food and Drug Administration (FDA) [3], a NS is a product taken by mouth that contains a “dietary ingredient”, which can be vitamin, mineral, herb, amino acid, enzyme or metabolite. NSs are generally offered both to prevent diabetes and support people with this disease.

NS use has gradually increased in both the United States (U.S.) [4-6] and Europe [7]. In the U.S. a programme called NHANES (National Health and Nutrition Examination Survey) has been developed to monitor the use of NSs in the population aged 1 year and older. Over the years, the age-adjusted prevalence of use of NS increased from 28% and 38% among adult males and females, respectively, in 1970–1974 to 44% and 53% in 2003–2006 [6]. In addition, a survey carried out in U.S. has shown that 73% of the U.S. adult population used one or more NSs in the year prior the interview and 4% of them reported an adverse event [5].

In the U.S. the use of NSs is higher in women, the elderly and people with healthier lifestyles and diets respect to other persons, but also in persons with chronic diseases such as diabetes mellitus [8,9]. Another study found that the use of alternative medicine was significantly higher among people with diabetes [10]. The European Community has commissioned the Health and Consumers Department to monitor the NSs market in Europe. Data from a recent report [7] shows an increase in the use of NSs between 1997 and 2005, but with broad variations from country to country. Market growth in fact ranged from 20% in the United Kingdom (U.K.) to 219% in Poland [7].

In recent years, online sales have contributed to the growth in the use of NSs, and currently an estimated 4% of total NS sales were carried out on internet [11].

Considering the high prevalence of NS use and their easy access on the internet, the aim of the research was to investigate the type of information provided by websites selling NSs on the prevention and treatment of diabetes. The list of ingredients was also considered in order to analyse the existence of scientific evidence regarding their possible effects on diabetes.

## Methods

### Search strategy

**Figure 1:**
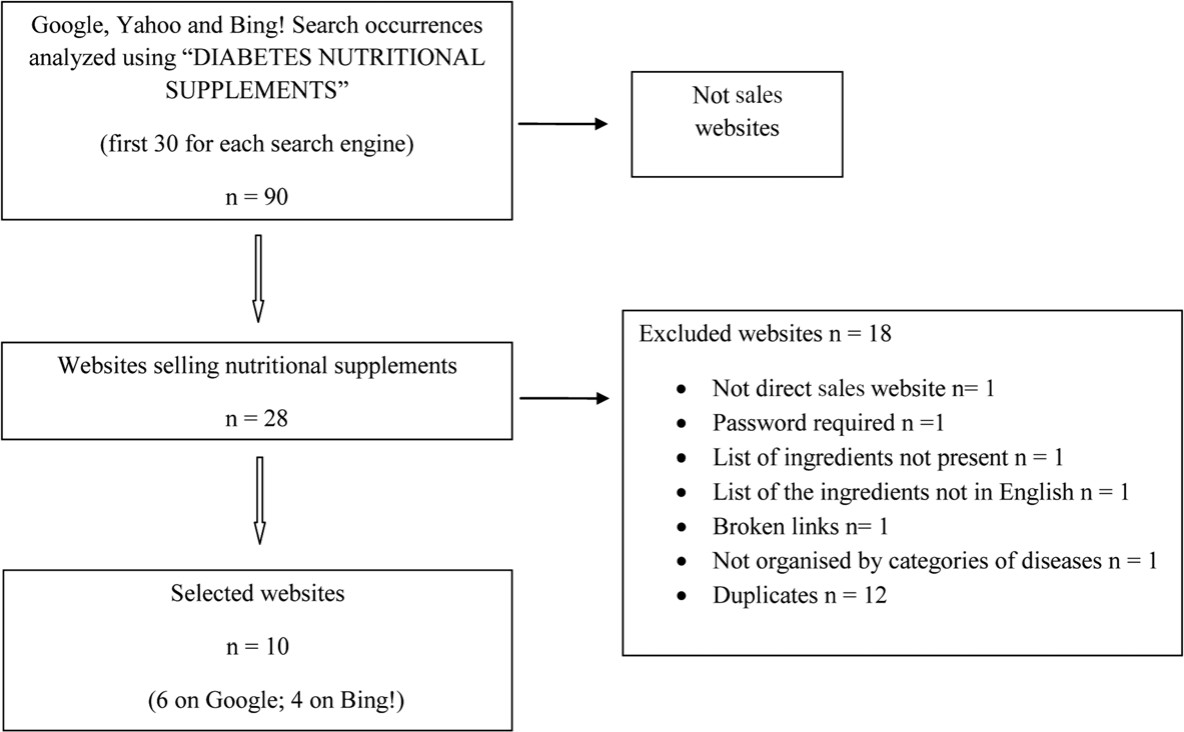
Diagram selection of web sites selling diabetes nutritional supplements.

The web search was conducted in April 2012, to identify websites selling NSs for the treatment and prevention of diabetes mellitus. The research was performed using 3 of the main search engines commonly used to seek information, Google, Yahoo and Bing! [12]. The key term used was “diabetes nutritional supplements”. As shown in Figure 1, the first 30 occurrences on the 3 search engines were analysed to identify websites selling NSs. Sites were included if (a) they sold supplements directly to the consumer, (b) they did not require a password to obtain the ingredients, (c) they showed the complete list of ingredients and (d) they were in English. Sites were excluded if they (a) were broken links and (b) were not organised by disease category. Twenty-eight websites were identified as sales websites; 12 of these were mentioned in more than one search engine and were only considered once. All the websites identified in Yahoo were the same identified in Bing!. Six ones didn’t fit the inclusion criteria. Only 10 sites suited our study purpose.

Under the “Diabetes” category we analysed the first nutritional supplement suggested by the website (the supplements were not shown in alphabetical order).

The content of each website was evaluated according to the main indications provided by the National Institute of Health Office of Dietary Supplements (NIH-ODS) on how to evaluate health information on the internet [13]. In particular, we evaluated how easy it was to find the person responsible for the website, the indication of the health information source and a possible reviewer of it, the presence of bibliographical references supporting the NS, and the presence of the FDA disclaimer statement. Testimonials, medical indications, safety claims and side effects provided by the site were also recorded.

### Literature review

In order to analyse scientific evidence of the efficacy of these supplements, a PubMed search was carried out on ingredients shared by at least 3 NSs. The bibliographical search for the specific ingredient was carried out, in addition to the following key words defined using the MeSH Database on PubMed: “Diabetes Mellitus” [Mesh]. The search was limited to randomized controlled trials (RCTs), systematic reviews (SRs) and meta-analyses (MAs) studies conducted on humans in the last 10 years.

The authoritative fact sheets on NSs provided by the NIH-ODS and the references reported by the sales websites in relation to the ingredients selected in this study were also considered. The FDA website was also used as a resource.

The literature analysis focused on the findings regarding the efficacy of these ingredients in treating diabetes mellitus, the method of administration, the indications for specific diabetes-related diseases and the possible side effects.

## Results

A total of 10 websites selling NSs for diabetes were analysed (Table 1). All the websites were located in USA. In general the NSs were offered to promote healthy blood sugar levels and reduce the risk of diabetes and health-related conditions. Half of them suggested contacting a physician about taking NS, two of them only in the case of pregnancy, breastfeeding or suspected medical problems. Four websites contained the FDA disclaimer “This statement has not been evaluated by the FDA. This product is not intended to diagnose, treat, cure, or prevent any disease”. On one website the FDA disclaimer was not complete. The company Xtend-Life stated that the product on offer was not a substitute for diabetes medication substitution, but this statement was in the FAQ section and there was no reference to the FDA. In only 3 out of 10 websites the manufacturer was clearly indicated. (Data not shown in table).

**Table 1:** Characteristics of nutritional supplements sales websites included in the study

**Company**	URL*	**Website location**	**Nutritional supplement**	**Health Professional contact suggested**	**FDA disclaimer†**	**Scientific references**	**Testimonials**
Type II Free	http://www.typefreediabetes.com	USA-New York	Glucocin	No	No	No	No
Xtendlife	http://www.xtend-life.com	USA-Georgia	Diabet-Eze	Yes	No‡	Yes	Yes
ADW American Diabetes wholesale	http://www.americandiabeteswholesale.com	USA-Florida	Diachieve Sugar Defense	No	No	Yes	Yes
Green Turtle Bay Vitamin Company	http://www.energywave.com	USA-Winsconsin	Diabetiks	Yes	Yes	No	Yes
Native Remedies	http://www.nativeremedies.com	USA-Texas	Insulate Plus	No	No	Yes	Yes
Heart, Diabetes & Weight Loss	http://www.vagnini.com	USA-Texas	AGE Essential Defence	No	No	No	No
NutriVera Naturals	http://www.nutrivera.com	USA-Minnesota	Alpha Betic	Yes	Yes	No	No
Optimum Diabetics	http://www.optimumdiabetics.com	USA-Texas	Optimum Diabetics	Yes	Yes	No	Yes
Get Healthy Again	http://www.gethealthyagain.com	USA-Florida	Custom Elixir D	No	No	No	No
Nutrient Synergy, Inc.	http://www.nutrientsynergy.com	USA-Florida	Nepretin	Yes	No§	No	Yes

*latest access on 20th April 2012 †FDA disclaimer: “This statement has not been evaluated by the FDA. This product is not intended to diagnose, treat, cure, or prevent any disease” ‡the statement that the product is not a diabetes medication replacement is contained in the FAQ section. §the statement was not complete.

Scientific references supporting the NS offered were present on 3 websites only. The characteristics of references retrieved on the three websites are shown in Table 2. Sixty percent of the websites selected showed testimonials from customers who had bought the NS.

**Table 2:** Characteristics of references retrieved on the websites selling nutritional supplements

**Nutritional supplement**	**Ingredient**	**N° of references**	N° of relevant references*	**Publication date of relevant references**
Glucocin				
	Biotin	19	2	1990
	Magnesium	26	2	1998
	Chromium	26	10	from 1987 to 2000
	*Momordica c.*	3	1	2007
	*Gymnema s*.	3	-	-
	*Camellia s.*	27	-	-
Diachieve				
Sugar Defense	†	23	3	1997/1999/2000
Insulate Plus				
	Chromium	2	1	1990
	*Gymnema s*.	1	-	-
	*Vaccinium m.*	2	1	1997

*studies carried out on humans and regarding diabetes, †The studies regarded the entire product and not the single ingredient.

**Figure 2:**
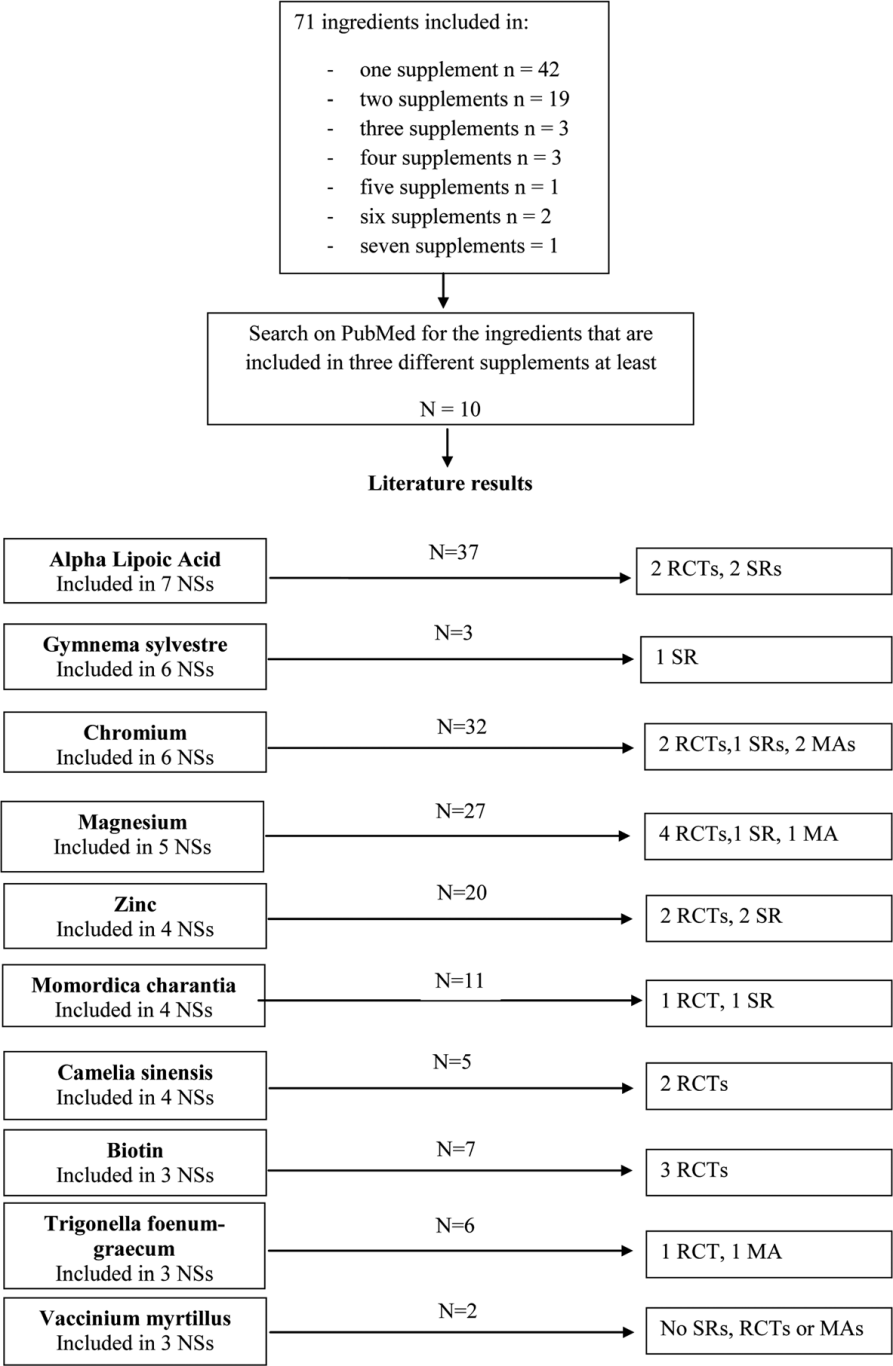
**Research on PubMed regarding the ingredients of nutritional supplements (NSs).** MAs, meta-analyses; NSs, nutritional supplements; RCTs, randomized control trials; SRs, systematic reviews.

We found a total of 71 different ingredients in the 10 NSs selected and some of them were present in more than one supplement (Figure 2).

A total of 10 NS ingredients were searched for on PubMed as they were present in at least 3 NSs. All the ingredients except two (*Gymnema sylvestre* and *Vaccinium myrtillus*) were analysed in RCTs (Figure 2).

In Additional file 1 are shown the characteristics of the studies investigating the ingredients selected and the main results. The largest RCT enrolled 447 subjects and regarded biotin. In the majority of cases (58%), the MA or SR retrieved only included a few studies (from 2 to 4).The largest study was an SR on chromium that encompassed 20 separate studies.

Reduction in fasting blood glucose, the most commonly used outcome measure, was statistically significant (p <; 0.05) for *Gymnema sylvestre* and alpha lipoic acid (ALA) in one of four studies, magnesium in two of three studies, chromium in four of six studies, zinc in one of four studies, biotin and *Momordica charantia* in one of three studies and *Trigonella foenum-graecum* in one of two studies.

Another outcome taken into account was the reduction in haemoglobin A1c, which was statistically significant (p <; 0.05) for *Gymnema sylvestre*, magnesium in one of two studies, chromium picolinate in one of four studies, zinc in one of two studies, biotin and *Trigonella foenum-graecum* in one study and *Momordica charantia* in one of two studies.

Adverse effects were rarely considered: in two of four studies for ALA, one of two studies for *Camellia sinensis*, two of three studies for biotin and *Momordica charantia* and one of two studies for *Trigonella foenum-graecum*.

Table 3 shows the dosage of the selected ingredient indicated in the NS offered by the websites and the tested dosage from the studies retrieved. For seven out of ten ingredients the dosage was specified on less than 50% of the websites. The daily dosage indicated for the NS offered by the websites and the dosage of the relative ingredient tested were comparable in the case of ALA, magnesium, chromium and zinc. In the other cases the dosages were quite different.

**Table 3:** Dosage recommended for nutritional supplements (NSs) offered by the companies online and dosage tested

**Ingredient**	**N° of websites on which the dosage was specified/websites selling the relative NS**	**Dosage per day (mg) indicated in the website**	**Dosage tested (mg)**	**Reference**
Alpha Lypoic Acid	1/6	600	300	Ansar et al., 2011 [14]
			600	de Oliveira, 2011 [15]
			600/1200/1800	Bartlett, 2008 [16]
			600/1200/1800	Lee, 2011 [17]
*Gymnema sylvestre*	1/6	2700	400	Leach, 2007 [18]
Magnesium	3/5	300 (2 websites)	365	Mooren, 2011[19]
		768 (1 website)	291.6 – 745.2	Bartlett, 2008 [17]
Chromium	2/5	0.3	0.02 – 1	Bartlett, 2008 [16]
		0.66	0.04	Sharma, 2011[20]
			0.5	Kròl, 2011[21]
			0.5 – 1	Ali, 2011[22]
			1	Cefalu, 2010 [23]
Zinc	3/4	15	10	Shidfar et al., 2010 [24]
		54	30	Bartlett, 2008 [16]
		78	50	Hussain, 2006 [25]
*Camellia sinensis*	2/4	40	150 – 600	Neyestani, 2010 [26]
		1800	375 – 750	MacKenzie, 2007 [27]
Biotin	2/3	0.2	2	Albarracin, 2008 [28]
			2	Geohas, 2007 [29]
			5	Revilla-Monsalve, 2006 [30]
*Momordica charantia*	1/3	2700	1000	Ooi, 2010 [31]
			500-2000	Fuangchan, 2011[32]
*Trigonella foenum-graecum*	0/3	-	2800	Losso, 2009 [33]
*Vaccinium myrtillus*	1/3	100	-	-

## Discussion

This study provides an overview of NSs for the prevention and treatment of diabetes available on the internet, and focuses on the type of information provided by the websites as well as the scientific evidence supporting these products.

NSs have gained in popularity in recent years and their use by the population has increased considerably because they are readily available and are considered “natural” substances that can improve or prevent a number of conditions [34]. The internet, as both a source of information and a marketing tool, has contributed to the easy accessibility of these products [11].

We analysed the content of 10 websites selling NSs for the treatment and prevention of diabetes, based on some of the NIH-ODS [13] and FDA indications.

In general, adequate medical information was lacking. Although NSs do not require FDA approval of their safety and efficacy, they are products offered to support people with diabetes and even mitigate the risk of disease-related complications in addition to prevent diabetes. For this reason, websites should take particular care when providing information. It should be noted that potential users, such as people with a chronic disease, already take drugs regularly. The risk of drug-NS interactions should not in fact be ruled out [35,36]. Only one website mentioned the possibility of side effects, and none mentioned possible interaction with drugs.

Additional concern could arise from the possibility of counterfeit products available on the Internet. NSs are less regulated than drugs and therefore more easily subject to counterfeiting [37]. As stated by FDA the manufacturer should be easily traced to guarantee transparency of the product [3]. It is interesting to note the manufacturer was clearly indicated in only three out of ten NS selected in this study but one of these one was not found in the list of manufacturers of the Dietary Supplements Labels Database [38].

It should be emphasised that NSs are not a substitute for medical products, but only half of the websites stated this clearly, four via the FDA disclaimer and one in the FAQ section. It is interesting to note that when this was specified, the testimonials could be misleading. For instance, a company claimed in the FAQ section that the NS was not a drug substitute, although one customer reported: “*…Today, his blood sugars are more stable… so much so that the doctor wants him to start weaning him off of his insulin…*”. Another company reported that the NS is a non-pharmaceutical product, without specifying that it is not intended to diagnose, cure, mitigate, treat, or prevent the disease. The testimonials in this website said such things as “*Thank you so much for saving my eyes. I have not had any surgeries in a long time*”, or “*My mom’s advancing eye disease stopped, and slightly reversed, and her kidney improvement is amazing*”. One of the NSs considered in this study was cited in a warning letter by the FDA in 2006 because it was in violation of Federal Food, Drug and Cosmetic Act [39] regarding a claim that should not have been included on the website as a testimonial “*…I had major problems with my feet, and always felt tingling and discomfort… Since using Insulate Plus this is a thing of the past…*”. This claim still remains, however.

It should be also noted that FDA states that a NS marker can make no health claims unless approved and that it is illegal to sell a NS and promote it on its label or in labelling as a treatment, prevention or cure for a specific disease or condition [3]. In our opinion some sentences retrieved in sales websites like *“Helps you prevent and care diabetes”* or “*helps block diabetes complications*” could be ambiguous.

These are many the reasons why it is necessary to consult a physician before taking a NS, but only five websites suggested contacting one, two of them only in the case of pregnancy, breastfeeding or suspected health problems.

This message should be presented clearly on the websites since it has been shown that only a small percentage of NS users (20-30%) inform their physician about supplement use [34,40]. Not informing their own physician, in addition to providing misleading information on the websites, could lead potential diabetic patients to stop taking conventional drugs, delay seeking adequate treatment as they are satisfied with their NS, or reduce adherence to conventional treatment [41]. Another point to consider is that potential users, such as patients with a chronic disease, could take these products for a long time. As far as we know, however, there is no information about the long-term effects of using NSs.

The risks for consumers regarding the misleading information provided by online companies selling heath related products direct to the consumer such as drugs [42] or genetic tests [43-45] are widely discussed by previous studies.

In the last years has been spreading the idea that people, in a view of a better prevention, need to be able to perform decision-making actions which are beneficial for the enhancement of their own health [46]. A recent study showed a great interest in complementary alternative medicine supplements in patients with diabetes as a strategy for active engagement in health and disease self-management [47]. It may be no coincidence that online companies often influence the formation of a positive attitude towards their products using the concept of empowerment. For instance sentences like “…*helps you manage your diabetes*…” or “*keeping diabetes management affordable*” found in some websites explain clearly this concept. However a misleading or not exhaustive information fails in making people empowered and a possible consequence is to lead people to a prevention not completely correct.

So it is also crucial that people acquire the ability to evaluate the information critically in order to be aware about their purchase and to use NS appropriately.

One of the NIH-ODS recommendations on how to evaluate health information on the internet [13] states that websites should provide medical and scientific evidence in support of the products presented. Testimonials by people claiming to have tried a particular product or service are not evidence-based and usually cannot be corroborated.

Only three websites provided scientific evidence supporting the NS offered. Few references actually concerned the effects on diabetes, however, and the majority of the articles were not recent (before 2002) (Table 2).

An analysis of literature in the last ten years about the most widely present ingredients found in NSs offered by the websites selected showed that scientific evidence is still lacking. There were RCTs and MAs or SRs for all but one of the ingredients selected, but the average number of subjects included in the majority of RCTs is 43 and the average number of studies included in MAs or SRs is 7. There is therefore a clear lack of large studies, particular RCTs, and the results are conflicting. These findings are in agreement with several studies seeking scientific evidence regarding NSs and diabetes [36,48] that emphasise that a real effect on diabetes management is not yet been established.

It is also interesting to note that the daily dosage of ingredients present in the NS, where indicated, was not very comparable to the dosage tested in the studies retrieved. In some cases the two dosages differed considerably, as in the case of *Gymnema sylvestre*, *Camellia sinensis*, biotin and *Momordica charantia*.

To our knowledge this is the first study focusing on NSs for the prevention and treatment of diabetes that are sold directly to the consumer on the internet. Concerns about the presence on the market of products of dubious efficacy are compounded with concerns about the misleading information – or lack of it – on the selling websites. Another point to underlined is the lack of a possible mediator, particularly a health professional, in the purchase of these products.

Some limitations of the study are the low number of websites retrieved and the fact that they are only in English. It is also reasonable to assume that this number will increase, as shown by data regarding use of the internet as a source of health information [49]. It is also interesting to note that a recent report [50] showed that 53% of American adults age 65 and over use the internet. This is the very age group affected by chronic disease. It was also shown that NS use is high among older people in UK [51]. In our opinion, the possible risks arising from this scenario that have been discussed in this study should not be ignored.

Another limitation of this study is that our evaluation of the scientific evidence in support of NSs only took into account one ingredient present in more than three products. It should be noted, however, that it is difficult to conduct research on NSs because product composition varies considerably from manufacturer to manufacturer, and different products have been studied. The ingredients are present in combination with other different ingredients in the NS and even the dosage varies greatly. It is therefore necessary to study the efficacy of NSs on diabetes, not the single ingredients, especially in the case of products claiming to reduce the risk of a disease or health-related condition, since potentially adverse effects and drug interactions cannot be entirely ruled out.

## Conclusion

In conclusion, easy internet access to NSs lacking adequate medical information and strong scientific evidence is a public health concern, mainly considering that a misleading information could lead to an improper prevention especially in healthy people and improper management of a disease in people suffering from diabetes. It is necessary to develop appropriate and reliable information to enable consumers to make an informed decision about the product prior to purchasing it. Furthermore, health care providers should be more informed about these widely used products. It is also evident that more research to validate the efficacy and safety of these NSs is called for.

## Abbreviations

ALA: Alpha lipoic acid; NHANES: National Health and Nutrition Examination Survey; NIH-ODS: National Institute of Health Office of Dietary Supplements; NS: Nutritional supplement; FDA: Food and Drug Administration; MA: Meta-analysis; RCT: Randomized controlled trial; SR: Systematic review; T2D: Type 2 diabetes.

## Competing interests

The authors declare that they have no competing interests.

## Authors’ contributions

The article was conceived by all authors. LC took responsibility for drafting the article. MC and LC carried out the Web search and interpreting of the data. EC, DF, LC and UG had a substantial role in critical revision. All the authors have given their final approval of the version for publication.

## Pre-publication history

The pre-publication history for this paper can be accessed here:

http://www.biomedcentral.com/1471-2458/13/777/prepub

## Supplementary Material

Additional file 1 **Characteristics of references retrieved on the websites selling nutritional supplements**[14-33,52-57]. Click here for file
