# Supplementary material for: Nutritional supplements for diabetes sold on the internet: business or health promotion?
Source: BMC Public Health. 2013 Aug 26;13:777. doi: 10.1186/1471-2458-13-777 (PMC3766237; doi:10.1186/1471-2458-13-777)
Supplement: Additional file 1 — Characteristics of references retrieved on the websites selling nutritional supplements[14-33,52-57]. [file 1471-2458-13-777-S1.pdf]

Additional file 1: Results of scientific evidence about the most reported ingredients on nutritional supplements on diabetes.

Ingredient	Reference	Type	N° of patients	N° of studies	Pharmaceutical formulation	Patients	Outcome measures	Significant results	Adverse effects
Alpha Lipoic Acid	Bartlett et Eperjesi, 2008 [32]	SR	-	2	ALA	Type 2 DM	FBG<br>Insulin resistance		Well tolerated
	Lee et Dugoua, 2011 [33]	SR	-	4	ALA	Type 2 DM	TSS<br>NSC<br>NIS	Improvement of neuropathic symptoms	Nausea, Vertigo and Vomiting for oral dose of ALA (1200/1800 mg)
	de Oliveira et al., 2011 [34]	RCT	102	-	ALA	Type 2 DM	Insulin resistance<br>FBG		NC
	Ansar et al., 2011 [35]	RCT	57	-	ALA	Type 2 DM	FBG<br>PPG<br>FSI<br>Insulin resistance	↓<br>↓<br><br>↓	NC
Gymnema sylvestre	Leach, 2007 [36]	SR	-	2	G. sylvestre leaf extract	Type 1 and type 2 DM	FBG<br>PPG<br>$HbA1_C$	↓<br>↓<br>↓	NC
Magnesium*	Larsson et Wolk, 2007 [37]	MA	-	7	Mg intake	Nondiabetic individuals	Risk of type 2 diabetes	↓	NC
	Bartlett et Eperjesi, 2008 [32]	SR	-	3	Mg oxide<br>Mg pindolate	Type 2 DM	Glycaemic control<br>FBG<br>$HbA1_C$<br>Fructosamine	 <br><br>↓	NC
	Mooren et al., 2011 [38]	RCT	52	-	Mg-aspartate-hydrochloride	Type 2 DM	FBG<br>FSI<br>Glucose after 120 min OGTT<br>Insulin after 120 min OGTT	↓	NC
	Rodriguez-Moran et al, 2003 [39]	RCT	63	-	Mg solution	Type 2 DM	FBG<br>$HbA1_C$<br>Insulin resistance	↓<br>↓<br>↓	NC

	Dong et al., 2011 [40]	MA	-	13	Mg intake	Nondiabetic individuals	Risk of type 2 DM	↓	NC
Chromium*	Althius et al, 2002 [41]	MA	-	20	Cr yeast<br>Cr nicotinate<br>Cr picolinate	Diabetic and Non diabetic individuals	FBG<br>FSI<br>$HbA1_C$		NC
	Bartlett et Eperjesi, 2008 [32]	SR	-	16	Cr yeast<br>Cr pindolate	Type 2 DM	FBG<br>$HbA1_C$<br>PPG		NC
					Cr picolinate	Type 2 DM	$HbA1_C$<br>FBG<br>FSI<br>PPG	↓<br>↓<br>↓<br>↓	NC
					Cr chloride	Type 2 DM	Fructosamine<br>$HbA1_C$<br>FBG<br>FSI	↓<br>↓<br>↓<br>↓	NC
	Cefalu et al., 2010 [42]	RCT	137	-	Cr picolinate	Type 2 DM	Insulin resistance<br>FBG<br>$HbA1_C$		NC
	Ali et al., 2011 [43]	RCT	59	-	Cr picolinate	High risk for type 2 DM	FBG<br>$HbA1_C$<br>Glucose after 120 min OGTT<br>Insulin after 120 min OGTT		NC
	Kròl et al., 2011 [44]	RCT	20	-	Cr brewer's yeast	Type 2 DM	FBG<br>$HbA1_C$<br>FSI<br>Insulin resistance	↓<br>↓<br>↓	NC
	Sharma et al., 2011 [45]	RCT	40	-	Cr yeast	Type 2 DM	FBG<br>$HbA1_C$	↓<br>↓	NC
Zinc*	Hussain et al., 2006 [46]	RCT	46	-	Zn acetate and Melatonin	Type 2 DM	FBG<br>PPG<br>$HbA1_C$	↓<br>↓<br>↓	NC
	Beletate et al., 2007 [47]	SR	-	1	Zn	Normal glucose tolerant obese	FBG<br>FSI		NC

						women	Insulin resistance		
	Bartlett et Eperjesi, 2008 [32]	SR	-	2	Zn glutonate	Type 2 DM	FBG<br>$HbA1_C$		NC
	Shidfar et al., 2010 [48]	RCT	48	-	Zn and Vitamin A	Type 1 DM	FBG		NC
Camellia sinensis	MacKenzie et al., 2007 [49]	RCT	49	-	Teaflavin extract	Type 2 DM	$HbA1_C$		Profuse sweating<br>Systemic rash
	Neyestani et al., 2010 [50]	RCT	46	-	Black tea extract	Type 2 DM	$HbA1_C$<br>Insulin resistance		NC
Biotin	Albarracin et al., 2008 [51]	RCT	447	-	Cr picolinate and Biotin	Type 2 DM	FBG<br>$HbA1_C$<br>FSI	↓<br>↓	Well tolerated
	Geohas et al., 2007 [52]	RCT	36	-	Cr picolinate and Biotin	Type 2 DM<br>Obese	Glucose after 120 min OGTT<br>Fructosamine<br>FBG	↓<br><br>↓	Well tolerated
	Revilla-Monsalve et al., 2006 [53]	RCT	33	-	Biotin	Type 2 DM<br>Nondiabetic individuals	FBG<br>FSI		NC
Momordica charantia	Ooi et al., 2010 [54]	SR	-	3	Fruits and seed preparation	Type 2 DM	FBG<br>$HbA1_C$<br>PPG		NC
	Fuangchan et al., 2011 [55]	RCT	129	-	Leaf preparation	Type 2 DM	FBG<br>$HbA1_C$	↓<br>↓	Abdominal pain, Diarrhoea
					Dried powder of fruit pulp		Fructosamine<br>FBG<br>Glucose after 120 min OGTT	↓<br>↓ Only for 2000 mg	Increased appetite, Palpitation
Trigonella foenum-graecum	Losso et al., 2009 [56]	RCT	8	-	Fenugreek bread	Type 2 DM	Glucose after 120 min OGTT<br>Insulin after 120 min OGTT	 ↓	Well tolerated
	Suksomboon et al., 2011 [57]	MA	-	9	Fenugreek extract	Type 2 DM	FBG<br>$HbA1_C$	↓	NC
Vaccinium myrtillus	-	-	-	-	-	-	-	-	-

DM: diabetes mellitus; ALA: Alpha Lipoic Acid; RCT: Randomized Controlled Trial; SR: Systematic Review; MA: Meta-analysis; FBG: Fasting Blood Glucose; PPG: Postprandial Blood Glucose; FSI: Fasting Serum Insulin; OGTT: Oral Glucose Tolerance Test; $HbA1_C$: Haemoglobin $A1_C$; TSS: Total Symptom Score; NSC: Neuropathy Symptoms and Change score; NIS: Neuropathy Impairment Score;↓: decreased statistically significant ($p<0.05$); NC: Not Considered

* Included in dietary supplementary fact sheets of NIH-ODS.
